# HIV-1 Matrix Dependent Membrane Targeting Is Regulated by Gag mRNA Trafficking

**DOI:** 10.1371/journal.pone.0006551

**Published:** 2009-08-07

**Authors:** Jing Jin, Timothy Sturgeon, Ora A. Weisz, Walther Mothes, Ronald C. Montelaro

**Affiliations:** 1 Department of Microbiology and Molecular Genetics, University of Pittsburgh, Pittsburgh, Pennsylvania, United States of America; 2 Renal-Electrolyte Division, Department of Medicine, University of Pittsburgh, Pittsburgh, Pennsylvania, United States of America; 3 Department of Cell Biology and Physiology, School of Medicine, University of Pittsburgh, Pittsburgh, Pennsylvania, United States of America; 4 Department of Infectious Disease and Microbiology, Graduate School of Public Health, University of Pittsburgh, Pittsburgh, Pennsylvania, United States of America; 5 Section of Microbial Pathogenesis, School of Medicine, Yale University, New Haven, Connecticut, United States of America; University of Minnesota, United States of America

## Abstract

Retroviral Gag polyproteins are necessary and sufficient for virus budding. Productive HIV-1 Gag assembly takes place at the plasma membrane. However, little is known about the mechanisms by which thousands of Gag molecules are targeted to the plasma membrane. Using a bimolecular fluorescence complementation (BiFC) assay, we recently reported that the cellular sites and efficiency of HIV-1 Gag assembly depend on the precise pathway of Gag mRNA export from the nucleus, known to be mediated by Rev. Here we describe an assembly deficiency in human cells for HIV Gag whose expression depends on hepatitis B virus (HBV) post-transcriptional regulatory element (PRE) mediated-mRNA nuclear export. PRE-dependent HIV Gag expressed well in human cells, but assembled with slower kinetics, accumulated intracellularly, and failed to associate with a lipid raft compartment where the wild-type Rev-dependent HIV-1 Gag efficiently assembles. Surprisingly, assembly and budding of PRE-dependent HIV Gag in human cells could be rescued *in trans* by co-expression of Rev-dependent Gag that provides correct membrane targeting signals, or *in cis* by replacing HIV matrix (MA) with other membrane targeting domains. Taken together, our results demonstrate deficient membrane targeting of PRE-dependent HIV-1 Gag and suggest that HIV MA function is regulated by the trafficking pathway of the encoding mRNA.

## Introduction

Retrovirus assembly and budding is a highly concerted process mediated by largely undefined spatially- and temporally-regulated interactions between viral proteins and cellular factors. During the viral assembly process, thousands of copies of viral structural polyproteins multimerize to form virus particles *via* an energy-dependent, multi-step process. Expression of retroviral Gag polyprotein is generally sufficient for the assembly and release of non-infectious virus like particles (VLPs). The Gag polyprotein consists of matrix (MA), capsid (CA), nucleocapsid (NC), late domain, and spacer proteins and is cleaved into the distinct structural proteins upon virus maturation [1], [2]. These Gag domains orchestrate the major steps in virus assembly and budding (reviews [1], [2]). It is well established that HIV-1 Gag buds from the plasma membrane of T lymphocytes and some epithelial cell lines [1]–[5]. In contrast, the major histocompatibility complex (MHC) class II compartments or multivesicular bodies (MVBs) are apparently the sites of HIV-1 Gag accumulation and particle production in macrophages and dendritic cells [6]–[9]. However, recent studies indicate that in macrophage HIV-1 virions bud from invaginated plasma membranes [10], [11]. Little is known about the precise mechanisms by which thousands of copies of Gag molecules synthesized from ribosomes in the cytoplasm are transported to specific locations on the plasma membrane for assembly and budding. Consistent with results published by Malim and colleagues [12], [13], our recent work suggests that HIV-1 Gag assembly is regulated at a step as early as nuclear export of its encoding mRNA [14].

Retroviral Gag polyproteins are synthesized from an unspliced full-length viral genomic mRNA that requires specific regulatory factors for nuclear export. The HIV-1 genome contains a *cis*-acting RNA element known as the Rev-response element (RRE) that binds to a viral *trans*-acting protein (Rev). Rev binds to the nuclear export protein Crm1 which in turn binds to Ran, a small GTPase that shuttles between the nucleus and the cytoplasm. Some simple retroviruses, such as Mason-Pfizer monkey virus (M-PMV), contain *cis*-acting RNA export elements (constitutive transport elements or CTE) that do not require viral *trans*-acting factors and that function by interacting directly with cellular export factors NXF1/NXT [13]. Swanson *et al* [12] recently demonstrated that altering the RNA nuclear export element used by HIV-1 *gag-pol* mRNA from the RRE to the CTE resulted in efficient trafficking and assembly of Gag at cellular membranes in murine cells, which are notable for their inability to support HIV-1 assembly and budding [12], [15], [16]. Our recent study also demonstrated that one copy of the hepatitis B virus posttranscriptional regulatory element (PRE) could support a similar level of HIV-1 Gag expression compared with Rev-dependent Gag [14] and that HIV-1 Gag assembly and budding in mouse cells could be rescued by substitution of the Rev-dependent RNA nuclear export signal with PRE [14]. Interestingly, in human cells the PRE-dependent, Rev-independent HIV-1 Gag showed lower assembly efficiency and different assembly sites compared with Rev-dependent HIV-1 Gag [14]. These results support the model that RNA export pathway selection during Gag expression and assembly can affect the cytosolic fate or function of the HIV-1 Gag polyproteins. In the current study, we sought to define the determinants of inhibited PRE-dependent HIV-1 Gag assembly and budding in human cells and to test whether altering these determinants can alleviate this block.

## Results

### Distinct Intracellular Distribution and Assembly Kinetics of Rev-dependent and PRE-dependent HIV-1 Gag

**Figure 1:**
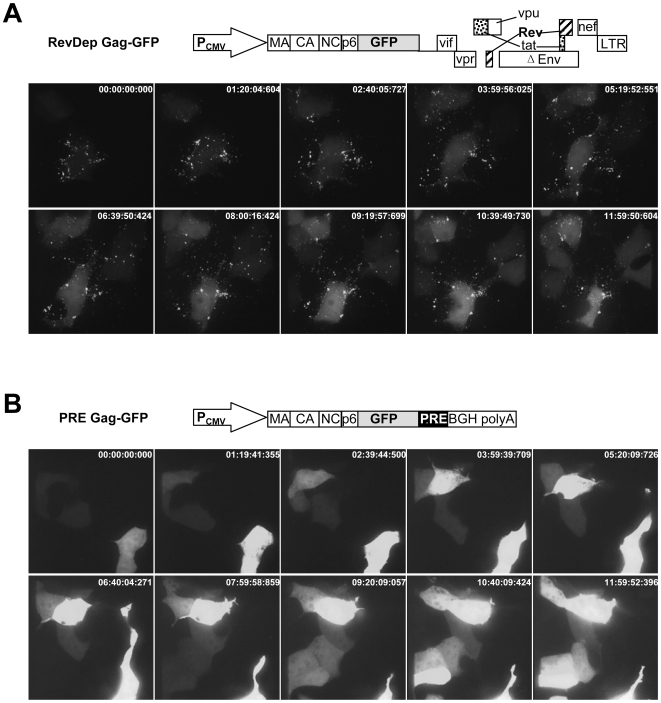
Distinct intracellular distribution and assembly dynamics of Rev-dependent and PRE-dependent HIV-1 Gag. 293T cells were transfected with Rev-dependent (A) and PRE-dependent (B) HIV-1 Gag-GFP and monitored with time-lapse confocal microscopy. Images of different Z sections were overlaid to produce single extended-focus image for each time point. Arrays of individual frames from two time-lapse videos covering about 12 hr compare the distribution and assembly dynamics of Rev-dependent and PRE-dependent HIV-1 Gag.

We recently demonstrated different assembly efficiencies and assembly sites for Rev-dependent and PRE-dependent HIV-1 Gag in both human and mouse cell lines [14]. We proposed that the observed distinct Gag assembly patterns could be the result of differential intracellular Gag trafficking as a consequence of the different pathways used for the export of HIV-1 Gag mRNA from the nucleus. Since the BiFC assays used in our previous study only revealed *assembled* Gag multimers inside cells, in the current study we first visualized the distribution of the total population of Rev-dependent or PRE-dependent HIV-1 Gag-GFP in 293T cells over time using live cell imaging. The results of these studies revealed that Rev-dependent HIV-1 Gag-GFP quickly (<1 hr) assembled into bright puncta on cell surface after appearance of the GFP signal (Fig. 1A and supplemental Video S1). In marked contrast, PRE-dependent HIV-1 Gag-GFP was diffusely distributed throughout the cells over a long time course (>10 hr), and did not form bright puncta on the cell surface even though large amounts of Gag-GFP were synthesized (Fig. 1B and supplemental Video S2). If the punctate Gag-GFP signal represents assembled Gag multimers as previously suggested [17], our results indicate faster assembly kinetics of Rev-dependent HIV-1 Gag compared with PRE-dependent HIV-1 Gag.

**Figure 2:**
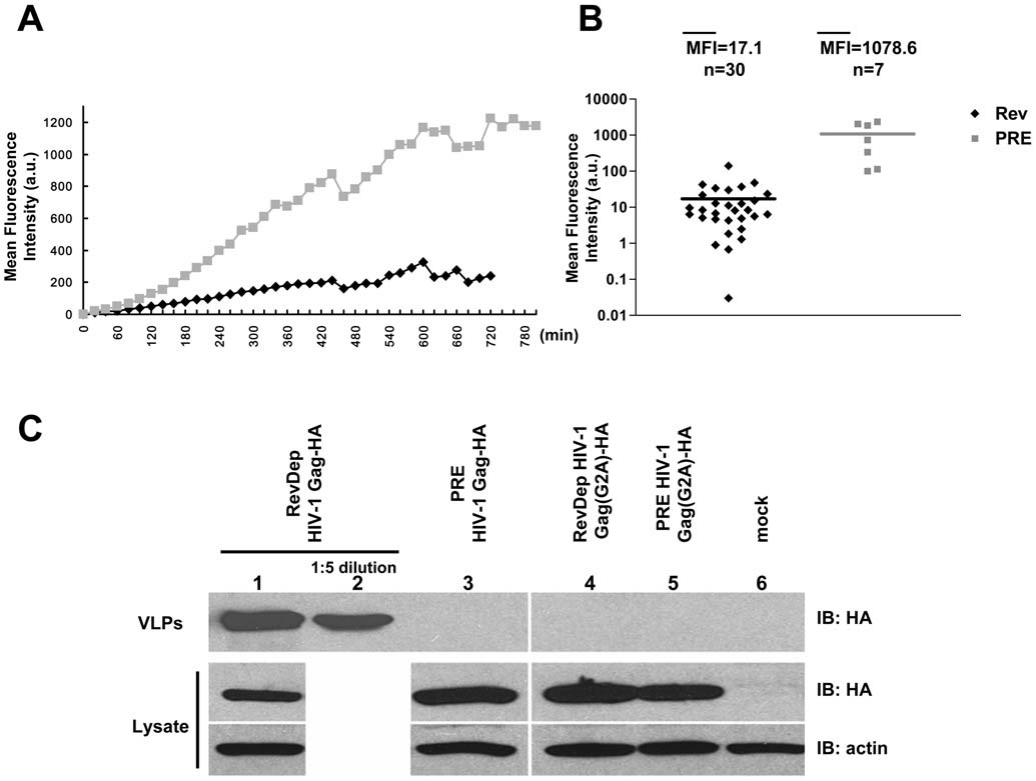
Deficient assembly and budding of PRE-dependent HIV-1 Gag. (A) Comparison of mean fluorescence intensity of 293T cells transfected with Rev-dependent (black diamond) or PRE-dependent HIV-1 Gag-GFP (gray square). Ceslls were monitored with time-lapse confocal microscopy as described in Figure 1. 30 randomly selected cells for each were tracked immediately after GFP fluorescence appeared and mean fluorescence intensity was measured. Average mean fluorescence intensity was plot over time. (B) Comparison of mean fluorescence intensity of 293T cells transfected with Rev-dependent (black diamond) or PRE-dependent HIV-1 Gag-GFP (gray square) when Gag-GFP puncta appear. All the 30 Rev-dependent HIV-1 Gag-GFP transfected cells tracked in (A) and 7 of the 30 PRE-dependent HIV-1 Gag-GFP transfected cells tracked in (A) displayed Gag-GFP puncta. (C) Budding of Rev-dependent and PRE-dependent wild type and myristoylation mutant HIV-1 Gag-HA. Rev-dependent and PRE-dependent Gag constructs were transfected into 293T cells. At 24 h post transfection, VLPs (upper panel) were analyzed by immunoblotting using HA antibody and cell lysates (lower panel) were analyzed by immunoblotting using HA and actin antibody. Data are representative of three independent experiments.

Recent reports indicate that the efficiency of retrovirus assembly depends on the concentration of Gag molecules [18]. To test if the observed delayed assembly of PRE-dependent HIV-1 Gag could be attributed to a lower expression level compared with Rev-dependent HIV-1 Gag, the expression and trafficking of Gag-GFP in individual cells was monitored by live cell imaging after transfection with either Rev-dependent or PRE-dependent HIV-1 Gag-GFP. For these assays, 30 cells from each transfection were randomly selected and analyzed individually, as summarized in Figure 2. These data reveal marked differences in the expression kinetics and trafficking patterns of the Gag-GFP produced by Rev-dependent or PRE-dependent expression. Both the Rev-dependent HIV-1 Gag-GFP expressing cells (black diamond) and the PRE-dependent HIV-1 Gag-GFP expressing cells (gray square) showed an increase of fluorescence over time (Figure 2A). However, the mean fluorescence intensity of PRE-dependent HIV-1 Gag-GFP expressing cells is consistently higher than Rev-dependent HIV-1 Gag-GFP expressing cells at all time points, with a 5-fold higher expression being observed at 9 hours after the initial detection of Gag-GFP expression. All the 30 randomly chosen Rev-dependent HIV-1 Gag-GFP expressing cells started to form bright puncta at surface at early time point (average 44 minutes after Gag-GFP started to be detected) with the average mean fluorescence intensity of 17 (Figure 2B), indicating a fast assembly kinetic of Rev-dependent HIV-1 Gag upon protein synthesis. In marked contrast, among the 30 randomly chosen PRE-dependent HIV-1 Gag-GFP expressing cells, only 7 cells displayed bright puncta at very late time points with the average mean fluorescence intensity of 1079 (Figure 2B). Taken together, this single cell imaging assay demonstrates that PRE-dependent Gag was in fact synthesized with similar kinetics and to similar concentrations as Rev-dependent Gag. However, the observations clearly indicated that the PRE-dependent Gag displayed a distinct trafficking pattern in transfected cells compared to the Rev-dependent Gag and failed to assemble into VLP as efficiently that leaded to intracellular Gag accumulation. Thus, these data support the concept that Gag assembly may be significantly influenced by the nuclear export pathway of its mRNA.

Having established similar expression levels in cells transfected with Rev-dependent or PRE-dependent Gag constructs, we next sought to compare the extracellular VLP production between cells transfected with either the Rev-dependent or PRE-dependent HIV-1 Gag. For these assays 293T cells were transfected with either HA-tagged PRE-dependent or Rev-dependent HIV-1 Gag expression plasmids. A G2A Gag mutant that lacks myristoylation and fails to bind to membranes for assembly was used as a reference for a budding deficient Gag [2]. Cells were transfected in parallel with Rev-dependent or PRE-dependent Gag, or with G2A mutant HIV-1 Gag expression plasmids. At 24 h post transfection, cell lysates and supernatant pellets (VLPs) were subjected to SDS-PAGE and Western Blotting to determine the respective Gag budding efficiencies (Figure 2C). The results indicate essentially undetectable levels of VLP budding from cells transfected with the PRE-dependent HIV-1 Gag, as observed with the myristoylation deficient G2A mutant Gag (Figure 2C, lines 3–5). In distinct contrast, a high level of extracellular VLP was produced from cells transfected with the Rev-dependent HIV-1 Gag, reflecting efficient assembly and budding by protein produced from this construct (Figure 2C, lines 1 and 2). Interestingly, analyses of the transfected cellular lysates indicated similar levels of intracellular Gag accumulation in cells transfected with any of the three assembly deficient Gag constructs, evidently reflecting similar levels of Gag expression regardless of the specific Gag construct (Figure 2C, compare line 3–5 and lines 1). Take together, these data are consistent with our previous report that Rev-dependent HIV-1 Gag assembles more efficiently in human cells compared with PRE-dependent HIV-1 Gag [14], despite the similar levels of Gag expression observed for the respective expression plasmids.

### Co-assembly of Rev-dependent and PRE-dependent HIV-1 Gag Rescues Budding of PRE-dependent HIV-1 Gag

**Figure 3:**
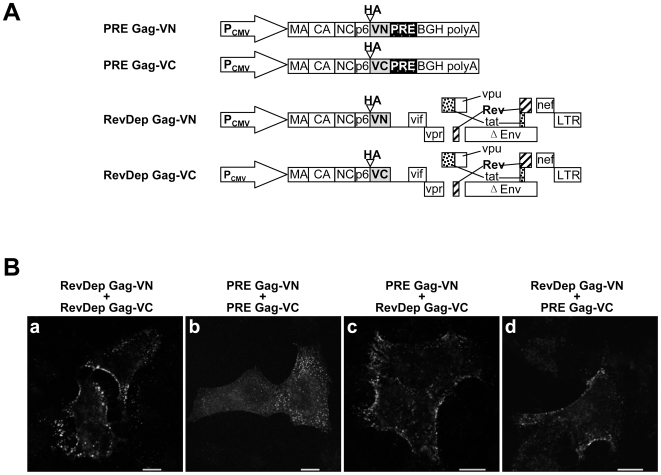
Co-assembly of Rev-dependent HIV-1 Gag and PRE-dependent HIV-1 Gag. (A) Schematic diagram of plasmids expressing Rev-dependent and PRE-dependent HIV-1 Gag-BiFC constructs. (B) Demonstration of co-assembly of Rev-dependent and PRE-dependent HIV-1 Gag by BiFC. HeLa cells grown on glass coverslips were transfected with plasmids expressing the indicated HIV-1 Gag-BiFC pair. At 8 h post transfection, cells were fixed and imaged. Bar: 10 µm.

We next took advantage of the BiFC assay to test whether Rev-dependent and PRE-dependent HIV-1 Gag can interact with each other even though they may utilize different trafficking pathways. Co-expression of Rev-dependent Gag-VC with PRE-dependent Gag-VN (Fig. 3B, panel c) or of Rev-dependent Gag-VN with PRE-dependent Gag-VC (Fig. 3B, panel d) both resulted in bright punctate BiFC signals on the plasma membrane, indicating interactions between Rev-dependent and PRE-dependent HIV-1 Gag. This pattern was similar to the BiFC pattern obtained upon coexpression of Rev-dependent HIV-1 Gag VN and VC pairs (Fig. 3B, panel a). In marked contrast, expression of PRE-dependent HIV-1 Gag VN and VC pairs resulted in a weaker intracellular punctate signal above a diffuse background (Fig. 3B, panel b), as we reported previously [14].

**Figure 4:**
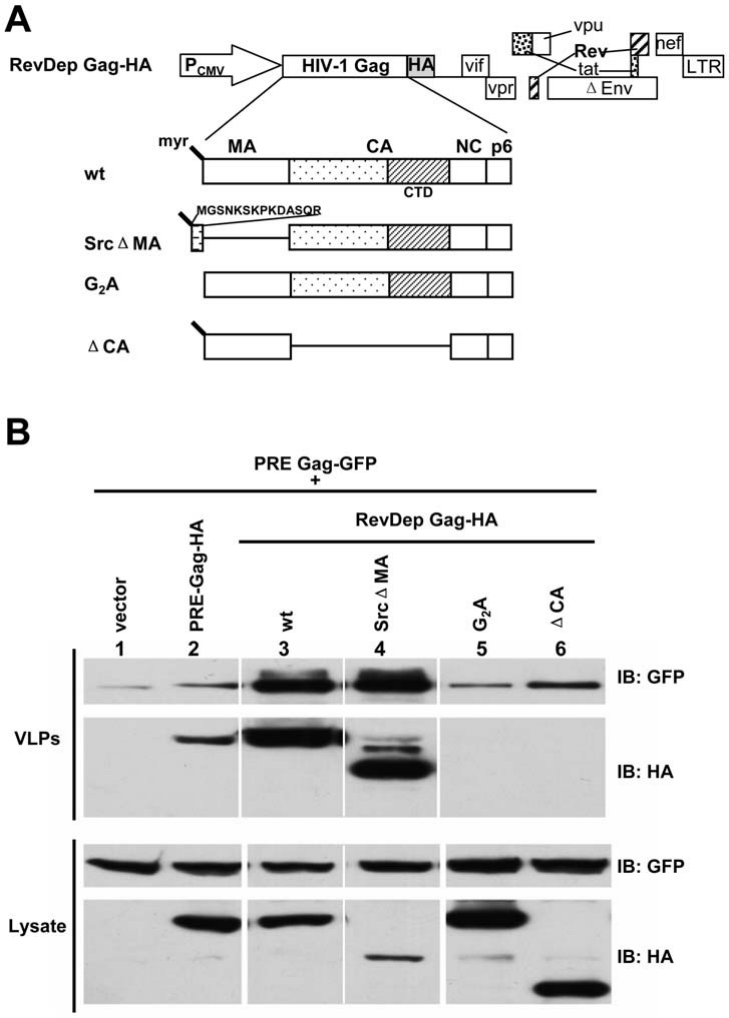
Coexpression of membrane targeting and assembly competent Rev-dependent HIV-1 Gag rescues PRE-dependent HIV-1 Gag budding. (A) Schematic diagram of plasmids expressing HA tagged Rev-dependent HIV-1 Gag mutants. (B) Budding of PRE-dependent HIV-1 Gag-GFP upon co-expression with PRE-dependent or Rev-dependent HIV-1 Gag-HA. PRE-dependent HIV-1 Gag-GFP was co-transfected at an equal molar ratio into 293T cells with empty vector, PRE-dependent HIV-1 Gag-HA, or the indicated Rev-dependent HIV-1 Gag-HA constructs described in A. At 24 h post transfection, VLPs (upper panel) and cell lysates (lower panel) were analyzed by immunoblotting using GFP and HA antibody. Data are representative of three independent experiments.

Since Rev-dependent HIV-1 Gag could efficiently co-assemble with PRE-dependent Gag at the plasma membrane, we next tested whether this co-assembly can rescue PRE-dependent HIV-1 Gag budding in human cells (Fig. 4B). PRE-dependent HIV-1 Gag-GFP was co-transfected with empty vector, HA-tagged PRE-dependent, or Rev-dependent HIV-1 Gag in 293T cells. At 24 h post transfection, cell lysates and supernatant pellets (VLPs) were subjected to SDS-PAGE and Western Blotting to determine budding efficiencies. Co-expression with Rev-dependent HIV-1 Gag-HA enhanced PRE-dependent HIV-1 Gag-GFP budding by about 30 fold (Fig. 4B, compare lanes 1 and 3). In contrast, this enhancement was not observed upon co-expression of PRE-dependent HIV-1 Gag-HA (Fig. 4B, compare lanes 1 and 2).

To gain further insight into the key determinants in Rev-dependent Gag required to restore budding of co-expressed Rev-independent Gag, we generated a panel of Rev-dependent HIV-1 Gag mutants (Fig. 4A) and tested their ability to rescue PRE-dependent HIV-1 Gag-GFP budding (Fig. 4B). A mutant with a constitutively exposed myristol residue generated by replacing MA with the myristoylation signal of v-Src (SrcΔMA) [19]–[21] (Fig. 4B, lane 4) could rescue budding of co-expressed PRE-dependent HIV-1 Gag-GFP as efficiently as wild type Rev-dependent HIV-1 Gag (Fig. 4B, lane 3). However, the myristoylation deficient G2A mutant (Fig. 4B, lane 5) and CA deletion mutant (ΔCA) (Fig. 4B, lane 6) were unable to enhance budding of co-expressed PRE-dependent HIV-1 Gag-GFP compared to co-expression of Rev-dependent wild type HIV-1 Gag (Fig. 4B, lane 2). Together, these results indicate that proper membrane association of Rev-dependent HIV-1 Gag constructs and co-assembly of Rev-dependent Gag with PRE-dependent Gag are required to rescue the budding of PRE-dependent HIV-1 Gag in human cells.

### Substitution of the Membrane Binding Domain Rescues PRE-dependent HIV-1 Gag Assembly and Budding

Because our results suggested that membrane targeting of Rev-dependent HIV-1 Gag is required to rescue budding of co-assembled PRE-dependent HIV-1 Gag, we next asked whether PRE-dependent HIV-1 Gag assembly and budding in human cells can be rescued by substitution of the HIV-1 membrane binding motif with other membrane targeting motifs. We have previously reported that in contrast to HIV-1 Gag, both Rev-dependent and PRE-dependent EIAV Gag can efficiently assemble and bud from human cells [14]. Thus, we also tested whether switching MA domains of EIAV and HIV-1 Gag altered their respective assembly and budding phenotypes.

**Figure 5:**
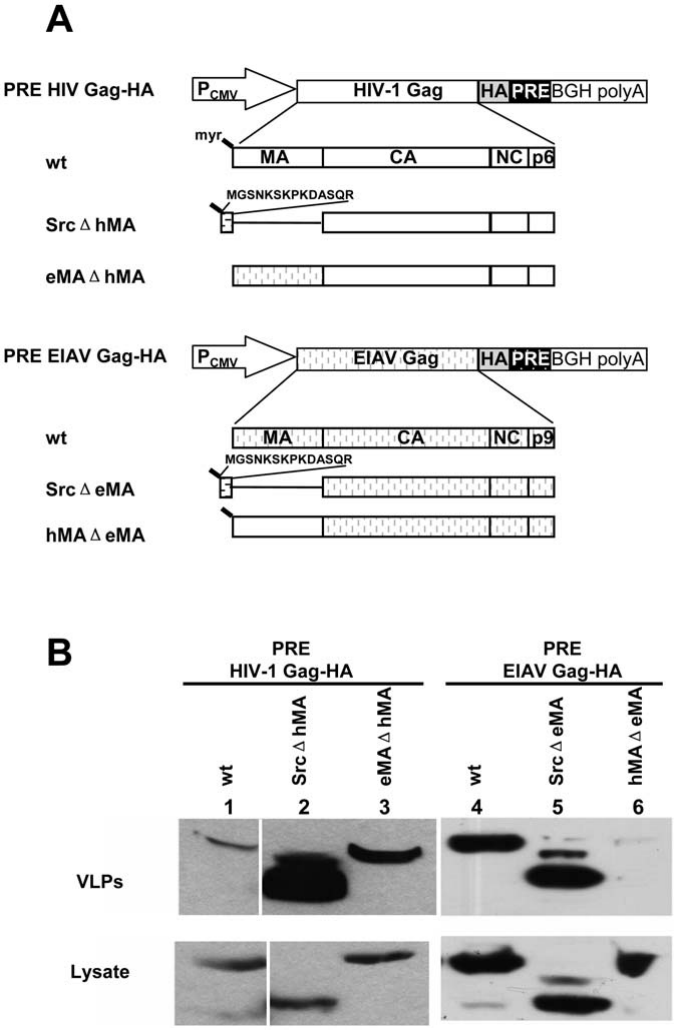
Substitution of HIV-1 matrix with other membrane targeting domains rescued PRE-dependent HIV-1 Gag budding in human cells. (A) Schematic diagram of plasmids expressing HA tagged PRE-dependent HIV-1 Gag mutants (upper panel) and EIAV Gag mutants (lower panel). (B) Budding of PRE-dependent HIV-1 and EIAV Gag mutants. 293T cells were transfected with the indicated PRE-dependent HIV-1 and EIAV Gag mutants described in (A). At 24 h post transfection, VLPs (upper panel) and cell lysates (lower panel) were analyzed by immunoblotting using HA antibody. Data are representative of three independent experiments.

We first constructed a panel of PRE-dependent HIV-1 Gag and EIAV Gag MA mutants (Fig. 5A). 293T cells were transfected with this panel of HA tagged PRE-dependent HIV-1 Gag and EIAV Gag constructs, followed by Western blotting of cell lysates and pelleted VLPs at 24 h post transfection. Replacing the HIV MA with the v-Src myristoylation signal rescued PRE-dependent HIV-1 Gag budding (Fig. 5B, compare lane 1 and lane 2). Consistent with our previous observations [14], and in contrast to PRE-dependent HIV-1 Gag, PRE-dependent EIAV Gag could bud efficiently from human cells (Fig. 5B, compare lane 1 and lane 4). Interestingly, MA swapping reversed this phenotype. PRE-dependent chimeric HIV-1 Gag containing EIAV MA budded as efficiently as PRE-dependent EIAV Gag (Fig. 5B, compare lane 3 and lane 5), whereas PRE-dependent chimeric EIAV Gag containing HIV MA failed to bud efficiently (Fig. 5B, lane 6). The PRE-dependent chimeric EIAV Gag budding deficiency seemed to be specific for the HIV MA, because replacing EIAV MA with the v-Src myristoylation signal did not interfere with chimeric EIAV Gag budding (Fig. 5B, lane 5).

### Distinct Membrane Association Properties of Rev-dependent and PRE-dependent HIV-1 Gag

**Figure 6:**
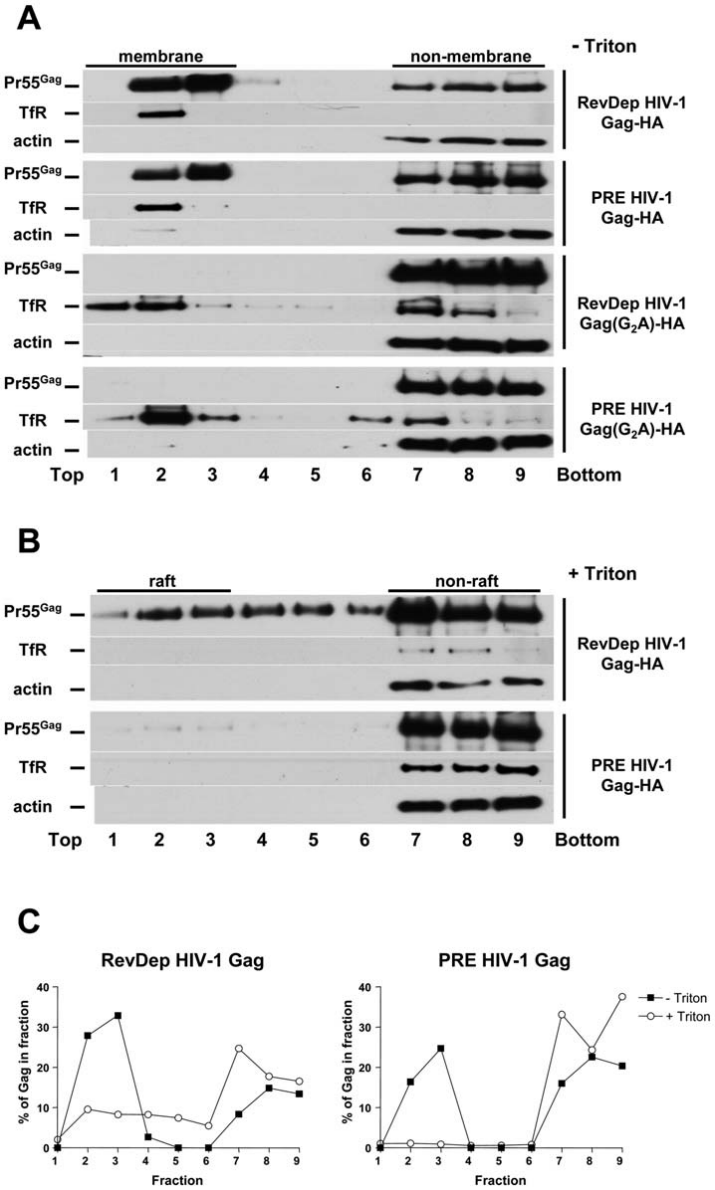
PRE-dependent HIV-1 Gag failed to associate with lipid raft in human cells. (A) Both Rev-dependent and PRE-dependent HIV-1 Gag are membrane-associated in human cells. Postnuclear supernatants derived from 293T cells expressing Rev-dependent or PRE-dependent HIV-1 Gag, Rev-dependent or PRE-dependent G2A mutant were subjected to equilibrium flotation centrifugation. Pr55^Gag^, TfR and actin were detected by Western blotting. Membrane- and non-membrane-associated fractions were shown. (B) Rev-dependent HIV-1 Gag but not PRE-dependent HIV-1 Gag is associated with Triton X-100 insoluble lipid rafts in human cells. Postnuclear supernatants derived from 293T cells expressing Rev-dependent or PRE-dependent HIV-1 Gag were treated with 0.5% Triton X-100 on ice for 30 min prior to membrane flotation analysis. Pr55^Gag^, TfR and actin were detected by Western blotting. Raft and non-raft fractions are indicated. (C) Quantitation of Rev-dependent (left panel) and PRE-dependent (right panel) HIV-1 Gag in each fraction with (open circles) or without (closed squares) cold Triton extraction.

Our live cell imaging results revealed an apparent cytoplasmic accumulation of PRE-dependent HIV-1 Gag over time. Additionally, the budding rescue assays described above suggested that MA-dependent membrane targeting of PRE-dependent HIV-1 Gag was apparently deficient in human cells. These observations led us to test whether the membrane association properties of PRE-dependent HIV-1 Gag are different from Rev-dependent HIV-1 Gag (Fig. 6). Postnuclear supernatants derived from 293T cells expressing either Rev-dependent HIV-1 Gag or PRE-dependent HIV-1 Gag were analyzed using membrane flotation to segregate membrane-associated and soluble Gag [22], [23]. After centrifugation, gradient fractions were collected and analyzed for Gag content by Western blotting. Transferrin receptor and actin were used as markers for membrane (fractions 1 to 3) and soluble (fractions 7 to 9) fractions, respectively. Roughly equal amounts of Rev-dependent (∼60%) and PRE-dependent (∼50%) HIV-1 Gag were recovered in membrane fractions, and these populations represented a significant proportion of the total Gag expressed in each case (Fig. 6A and 6C). Under the same conditions, myristoylation deficient G2A mutants were not present in membrane fractions regardless of whether they were expressed in a Rev-dependent or PRE-dependent context, consistent with previous reports [23]–[25]. These data suggest that PRE-dependent HIV-1 Gag can target human cell membranes and is unlikely to be myristoylation deficient, consistent with other studies showing efficient membrane association of other Rev-independent HIV-1 Gag in human cells [25]–[27]. Taken together with the fluorescence microscopy results (Fig. 1), our data suggest that unlike Rev-dependent HIV-1 Gag, PRE-dependent HIV-1 Gag fails to specifically bind to plasma membrane. Cytosolic soluble Gag and random membrane bound Gag together display a diffusive distribution pattern of PRE-dependent Gag in human cells.

It has been reported that plasma membrane lipid rafts play a critical role in HIV-1 Gag assembly and budding [28]–[33]. Raft association of HIV-1 Gag occurs after membrane binding [31] and is independent of the Gag assembly ability [30], [31]. Therefore we next tested whether Rev-dependent and PRE-dependent HIV-1 Gag were differentially targeted to lipid rafts (Fig. 6B). Postnuclear supernatants derived from 293T cells expressing either Rev-dependent HIV-1 Gag or PRE-dependent HIV-1 Gag were analyzed as described above except that they were treated with 0.5% Triton X-100 on ice for 30 min before being subjected to membrane flotation. Under these conditions, transferrin receptor, a membrane protein known not to partition into detergent-insoluble microdomains, was not detected in lipid raft fractions. About one third of the membrane-associated Rev-dependent HIV-1 Gag could still be detected in these fractions after cold Triton X-100 treatment, demonstrating its association with lipid rafts or detergent-resistant membranes (Fig. 6C, left panel). In contrast, only 6% of the membrane-associated Rev-independent HIV-1 Gag was present in lipid raft-containing fractions after detergent extraction (Fig. 6C, right panel).

## Discussion

Recently, we reported that HIV-1 Gag assembly and budding are regulated by the nuclear export pathway of Gag-encoding mRNA [14]. PRE-dependent HIV-1 Gag was trafficked differently as compared to native Rev-dependent Gag resulting in a defect in Gag assembly and budding in human cells [14]. In the current study, we performed a mechanistic analysis of the regulation of HIV-1 Gag assembly and budding by mRNA nuclear export pathways. We demonstrate that PRE-dependent HIV-1 Gag is mistargeted in human cells, evidently because the Gag produced by PRE-dependent expression lacks the membrane raft targeting function of HIV MA. This finding is reminiscent of deficient Rev-dependent HIV-1 Gag assembly/budding in mouse cells resulting from deficient MA dependent membrane targeting [12], [26], [34]. As such, the current data supports the concept that MA-directed membrane targeting and assembly of Gag is dependent on the pathway used for trafficking of the Gag mRNA.

It is thought that the primary function of the MA domain in retrovirus assembly is to mediate membrane association of Gag polyprotein. The three-dimensional structure of HIV-1 MA reveals a globular head conformation [35], [36]. The N-terminal myristic acid and the highly basic patch formed by conserved positive charged residues clustered on the surface of the MA globular head both contribute to HIV-1 MA dependent membrane binding of Gag precursors [37]. Structural studies demonstrate specific binding interactions between myristoylated HIV-1 MA and phosphatidylinositol-(4,5)-bisphosphate [PI(4,5)P2], which triggers a change in protein conformation that flips myristate from the sequestered to the exposed conformation, thereby promoting the stable association of MA with the membrane [20]. Previous intracellular functional studies also demonstrate that PI(4,5)P2 plays a key role in Gag targeting to the plasma membrane [4] and the recent lipid analysis of HIV-1 virions demonstrated a MA-dependent enrichment of PI(4,5)P2 in viral lipid envelope [28]. It has been suggested that PI(4,5)P2 may preferentially associate with lipid rafts [38], [39]. Therefore the PI(4,5)P2 induced myristoyl switch could regulate lateral targeting of PI(4,5)P2:Gag complexes to lipid rafts that play a critical role in HIV-1 assembly and budding [31]–[33], [40]. In addition to lipid, HIV-1 MA also binds to RNA through its basic residues [41], [42]. Therefore it can be speculated that RNA binding might place another layer of regulation on MA dependent membrane targeting, possibly by regulating the PI(4,5)P2-induced myristoyl switch of HIV-1 MA.

Our data suggest that HIV MA in the context of PRE-dependent Gag causes unspecific membrane targeting. Promiscuous membrane binding would prevent Gag association with plasma membrane lipid rafts that support Gag assembly. Our current studies do not address the detailed molecular mechanism behind the mistargeting of PRE-dependent HIV-1 Gag in human cells. At this time, we can only speculate as to how RNA export pathways affect the membrane targeting function of HIV-1 MA.

We hypothesize that HIV MA mediated plasma membrane rafts targeting is tightly regulated both temporally and spatially and relies on the sequential acquisition and release of host factors. In human cells, PRE-dependent Gag may exhibit a defect in the PI(4,5)P2 regulated myristoyl switch that could be due to the inability to associate with specific cellular cofactors (RNA or proteins). This defect can be rescued by *in trans* by Rev-dependent wild type HIV-1 Gag but not by myristoylation deficient G2A or multimerization deficient ΔCA mutants. Importantly, the deficiency of PRE-dependent HIV-1 Gag can also be rescued *in cis* by replacing MA with other membrane targeting motifs, suggesting that efficient membrane targeting by HIV MA requires Rev-dependent trafficking. In the absence of Rev-dependent trafficking, MA exhibits an inhibitory effect on Gag assembly. Retroviral genomic RNA (gRNA) serves as the mRNA template for Gag synthesis as well as the genetic component of infectious viruses. In our studies, the PRE-dependent HIV-1 Gag was expressed from a mRNA encoding no viral accessory proteins and containing no gRNA sequence. We believe that it is unlikely that the deficiency of PRE-dependent HIV-1 Gag would only be due to the lack of these viral factors, because membrane binding (G2A) and multimerization deficient (ΔCA) Rev-dependent Gag constructs failed to rescue PRE-dependent Gag assembly and budding, despite expressing all accessory proteins. In addition, although gRNA provides the scaffold for Gag assembly, this function can be replaced by cellular tRNA and rRNA [43].

Various studies in cell biology have demonstrated that specific RNA localization is a widely used mechanism affecting protein function at multiple levels [44]. For example, ∼70% of mRNAs in oocytes and early embryos of *Drosophila* are localized in dozens of distinct patterns [45]. Considerable evidence suggests that retroviral Gag trafficking and assembly/budding is regulated by both nuclear export [12]–[14], [46] and cytoplasmic transport [22], [47] of viral gRNA. Our results comparing trafficking and assembly of HIV-1 Gag expressed from mRNA using Rev-dependent (Crm 1 dependent) [48] and PRE-dependent (Crm 1 independent) [49] nuclear export pathways provide further support for this idea. At this point, we do not know whether localized Gag synthesis plays a role in plasma membrane targeting or if Gag synthesized from RNA takes different trafficking routes. Clearly, the current data argue that RNA trafficking also regulates protein function during retrovirus assembly. In closing, PRE-dependent HIV-1 Gag exhibits an MA-dependent assembly defect in human cells. Together with assembly deficient Rev-dependent HIV-1 Gag in mouse cells, this model can provide a valuable tool to study how HIV assembly is temporally and spatially regulated and coordinated with genome packing.

## Materials and Methods

### DNA mutagenesis

Overlapping PCR was used to construct Gag mutations and fusion proteins. Rev-dependent and Rev-independent HIV-1 (pNL4–3 proviral clone [50]) and EIAV (pEIAVuk proviral clone [51]) Gag expression vectors were described previously [14]. Briefly, the hepatitis B virus posttranscriptional regulatory element (PRE) was attached to the C-terminus of EIAV or HIV-1 Gag gene to generate Rev-independent EIAV or HIV-1 Gag expression vectors. Rev-dependent EIAV or HIV-1 Gag constructs were made based on pEIAVuk or pNL4–3 proviral constructs. For BiFC assays, gene sequences encoding the amino (residues 1–173, VN)- or carboxyl (residues 155–238, VC)- fragments of Venus fluorescence protein were fused to the C-terminus of EIAV or HIV-1 Gag via a 6-alanine linker as described previously [14]. To make hemagglutinin (HA) epitope-tagged Gag polyproteins, the YPYDVPDYA epitope from influenza virus HA protein was inserted into the C-terminus of p9 or p6 protein_,_ respectively. All plasmids were isolated using the Qiagen Midiprep Kit (Qiagen, Valencia, CA), and the specific mutations were confirmed by DNA sequencing.

### Cell culture and transfection

HeLa SS6 and 293T cells were cultured in Dulbecco's Modified Essential Medium (DMEM) supplemented with 10% fetal bovine serum (Invitrogen, Carlsbad, CA). Cells were transfected using Lipofectamine 2000 (Invitrogen, Carlsbad, CA) following the procedures outlined by the manufacturer.

### Gag budding assays

At 24 h post transfection, cells were harvested and lysed in lysis buffer (25 mM Tris-HCl, pH 8.0, 150 mM NaCl, 1% deoxycholic acid, 1% Triton X-100, 1× protease inhibitor cocktail) and centrifuged at 20,800×*g* for 5 min to remove cell nuclei. Virus-like particles (VLPs) released into the culture medium were pelleted by centrifugation (20,800×g for 3 h at 4°C) and resuspended in PBS. HA-Gag contained in cell lysates and VLPs was analyzed by Western Blotting using rat anti-HA antibody (Roche Applied Science, Indianapolis, IN) and HRP conjugated goat anti-rat IgG (Invitrogen, Carlsbad, CA), as described previously [14].

### Membrane flotation analysis

Membrane flotation procedures were performed as described [22], [23]. At 24 h post-transfection, 293T cells were washed twice with phosphate-buffered saline, collected, and homogenized in 300 µl of TE buffer (10 mM Tris [pH 7.4], 1 mM EDTA [pH 8]) containing 10% sucrose and protease inhibitor cocktail by passaging 24 times through a 23G11/4 needle. Nuclei were removed by centrifugation at 1,000×*g*. A 250 µl sample of post nuclear supernatant (PNS) was mixed with 1.25 ml of TE 85.5% sucrose (adjusting the concentration of sucrose to 73%) and deposited at the bottom of a 5-ml centrifugation tube. TE 65% sucrose (2.5 ml) and then 1 ml of TE 10% sucrose were layered above the lysate. The samples were subjected to ultracentrifugation at 100,000×*g* for at least 14 h at 4°C in a SW55Ti rotor (Beckman Coulter). To determine lipid raft association, PNSs were treated with Triton X-100 (final concentration 0.5%) for 30 min on ice prior to flotation centrifugation. Nine fractions of 550 µl were collected from the top and analyzed by Western Blotting using the rat monoclonal anti-HA antibody (Roche Applied Science, Indianapolis, IN), mouse monoclonal anti-human transferrin receptor antibody (Invitrogen, Carlsbad, CA) and mouse monoclonal anti-βactin antibody (Sigma-Aldrich, Saint Louis, MO). X-ray films were scanned and analyzed by the ImageJ 1.38× software (http://rsb.info.nih.gov/ij/).

### Imaging

For BiFC assays, transfected cells grown on coverslips were fixed and permeabilized with 2% paraformaldehyde and 0.1% Triton X-100 in PBS. Images were captured using a Leica TCS-SL microscope and processed with Metamorph software. For live cell imaging, cells were plated on 35-mm imaging dishes (MatTek, Ashland, MA) and transfected with GFP tagged HIV Gag expression vector. At 8 hr post transfection, images were captured every 20 min using a Nikon TE2000E spinning disc confocal microscope. Single cell tracking was performed using Volocity software tracking module (Improvision, Lexington, MA). All time-lapse movies were edited using Volocity software (Improvision, Lexington, MA).

## Supporting Information

Video S1 Intracellular distribution and assembly dynamics of Rev-dependent HIV-1 Gag. 293T cells were transfected with Rev-dependent HIV-1 Gag-GFP and monitored with time-lapse confocal microscopy from 6 hour post transfection. Three dimensional images were taken every 20 minutes. For each time point, images of different Z sections were overlaid to produce single extended-focus image.
(4.95 MB MOV)

Video S2 Intracellular distribution and assembly dynamics of PRE-dependent HIV-1 Gag. 293T cells were transfected with PRE-dependent HIV-1 Gag-GFP and monitored with time-lapse confocal microscopy from 6 hour post transfection. Three dimensional images were taken every 20 minutes. For each time point, images of different Z sections were overlaid to produce single extended-focus image.For each time point, images of different Z sections were overlaid to produce single extended-focus image.
(6.62 MB MOV)
